# Lipoid pneumonia presenting as non resolving community acquired pneumonia: a case report

**DOI:** 10.1186/1757-1626-2-9332

**Published:** 2009-12-16

**Authors:** Vijay Hadda, Gopi C Khilnani, Ashu S Bhalla, Sandeep Mathur

**Affiliations:** 1 Department of Medicine, All India Institute of Medical Sciences, New Delhi, 110029, India; 2 Department of Radio diagnosis, All India Institute of Medical Sciences, New Delhi, I110029, India; 3 Department of Pathology, All India Institute of Medical Sciences, New Delhi, 110029, India

## Abstract

**Introduction:**

Lipoid pneumonia is a rare form of pneumonia caused by inhalation or aspiration of fat containing substances like, petroleum jelly, mineral oils, few laxatives etc. It usually presents as insidious onset chronic respiratory illness simulating interstitial lung diseases. Rarely, it may present as an acute respiratory illness, specially, when exposure to fatty substance is acute and/or massive. Radiologically, it may mimic carcinoma, acute or chronic pneumonia, ARDS, or a localized granuloma. Diagnosis of LP requires demonstration of lipid laden macrophages in sputum, bronchoalveolar lavage fluid or fine needle aspiration cytology/biopsy from lung lesion. Treatment of this illness is poorly defined and constitutes supportive therapy and corticosteroids.

**Case presentation:**

A 20-year old Indian farmer was referred to us with a diagnosis of non resolving community acquired pneumonia. Respiratory examination revealed signs of consolidation. Chest radiograph revealed findings suggestive of bilateral consolidation. Sputum and blood culture were sterile. He was treated with prolonged course of various antibiotics without any significant response. For evaluation of non resolving pneumonia fibreoptic bronchoscopy was done. Bronchoalveolar lavage fluid and biopsy from lung lesion showed lipid laden macrophages. Hence diagnosis of lipoid pneumonia was made. Patient was treated with course of corticosteroids with good response. Literature on this rare entity is discussed.

**Conclusion:**

Lipoid pneumonia is a rare form of pneumonia which rarely present acutely resembling community acquired pneumonia and requires high degree of suspicion for diagnosis. Its treatment is difficult and poorly defined. However, prolonged corticosteroids may be effective.

## Introduction

Lipoid pneumonia (LP) is a rare form of pneumonia caused by inhalation or aspiration of a fatty substance. This entity was first described in initial half of twentieth century [1]. It usually presents as a chronic respiratory disease with symptoms of shortness of breath and cough. However, it may present as acute respiratory illness when exposure is to large quantity of mineral oil [2,3]. This entity does not have characteristic clinical or radiological features. Therefore, awareness of this type of pneumonia is important as diagnosis requires a high degree of clinical suspicion. It should be considered in the differential diagnoses of any acute or chronic respiratory condition in cases with history of exposure to fatty substances. The diagnosis of acute LP, in absence of history of exposure to fatty substances, may be very difficult. However, early diagnosis and timely treatment is important for better outcome. In suspected cases, diagnosis can be established by demonstrating lipid laden macrophages in various specimens like sputum, bronchoalveolar lavage, lung biopsy [4,5]. We present a case with acute LP with acute lung injury. The available literature on this rare entity is discussed.

## Case presentation

A 20 years old Indian farmer was referred to us from another hospital with a diagnosis of bilateral non resolving pneumonia. This patient was admitted in another hospital located about 250 kilometers from Delhi with complaints of acute onset high grade fever with chills, cough with expectoration, shortness of breath and bilateral pleuritic chest pain. There was no hemoptysis. He was treated in that hospital with antibiotics (details not available) and supportive therapy for 8 days without any response. He was referred to our hospital with diagnosis of non resolving community acquired pneumonia. He presented to our hospital with high grade fever, cough with profuse expectoration, dyspnea, and bilateral pleuritic chest pain. There was no history of bowel or urinary symptoms. On examination, patient was dyspneic and tachypneic with respiratory rate of 36/minute. He was febrile with temperature of 103°F. His pulse rate was 120/minute and blood pressure was 100/60 mm of Hg. There was no pallor, icterus, cyanosis, or lymphadenopathy. Respiratory system examination revealed bronchial breathing in interscapular and infrascapular region on left side of the chest. Along with it there were extensive bilateral coarse crepitations. Examination of other systems including cardiovascular, abdominal and nervous system was normal.

**Figure 1:**
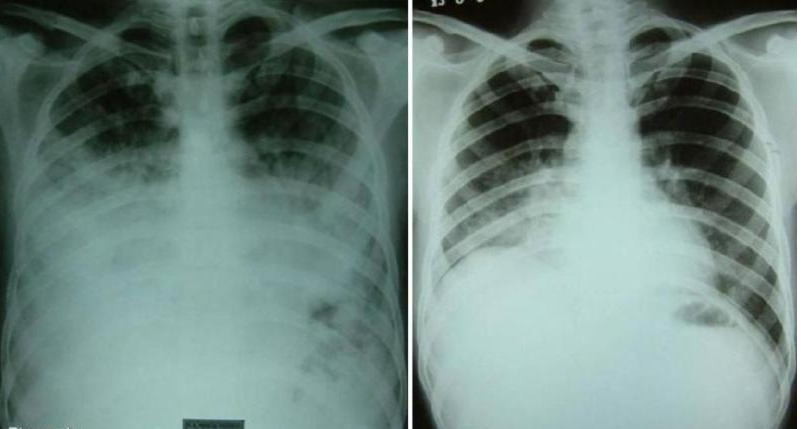
**Frontal chest radiograph (figure 1a) reveals extensive consolidation involving mid and lower zones of bilateral lung fields**. Follow up chest radiograph (figure 1b) obtained three months later shows significant clearing of consolidation.

Blood investigations revealed hemoglobin of 12.0 gm/dl, leukocyte counts of 21,400/dl with 89% neutrophils, and normal platelets (310 × 10^3^/dl). Arterial blood gas analysis on FiO_2 _of 0.3 revealed - pH of 7.4, pO_2 _of 70.4 mm of Hg, pCO_2 _of 37.6 mm of Hg and oxygen saturation (SaO_2_) of 94%. PaO_2_/FiO_2 _was 234.6 indicating acute lung injury. Blood urea (34 mg/dl) and creatinine (0.9 mg/dl) were normal. Liver function tests showed normal bilirubin and mild hepatitis with SGOT and SGPT of 90 and 53 IU, respectively. Blood and sputum cultures were negative for any growth. X-ray chest revealed bilateral middle and lower zone consolidation (Figure 1a).

**Figure 2:**
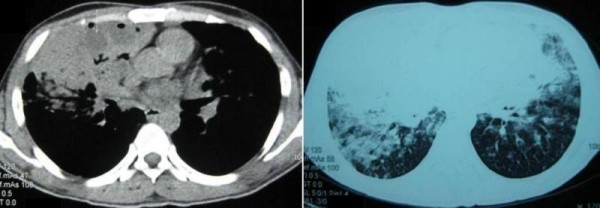
**Mediastinal (Figure 2a) and lung window (figure 2b) of non-contrast computer tomographic scan of chest reveals consolidation in the right middle lobe, lower lobe, and also left lung**. Few low density areas (arrow) are seen within the middle lobe consolidation.

**Figure 3:**
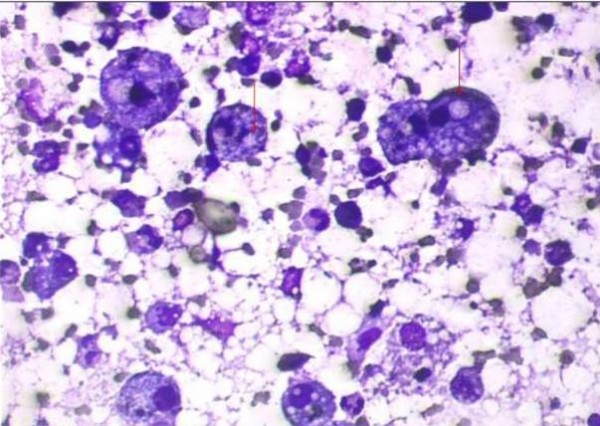
**Bronchoalveolar lavage fluid cytology showing lipid laden macrophages**.

He was treated with various intravenous (IV) antibiotics including piperacillin-tazobactum, azithromycin, cefoperazone-sulbactum, amikacin, and clindamycin. He also required oxygen supplementation with FiO_2 _of 0.3 to maintain his SaO_2 _in the range of 92 - 94%. Fever settled after six days of hospitalization, however, patient consistently had persistent crepitations, bronchial breathing, infiltrates on chest X-ray and raised total leukocyte count (TLC) in the range of 15400 to 26400/dl. Therefore, patient was subjected to computer tomographic (CT) scan of chest (Figure 2a and 2b) which revealed consolidation in the right middle lobe and lower lobe and also left lung. Few low density areas were seen within the right middle lobe. Patient underwent fibreoptic bronchoscopy (FOB) with bronchoalveolar lavage (BAL) and transbronchial lung biopsy (TBLB) from right lower lobe. FOB showed normal airways. BAL fluid was sterile on culture. Staining and culture for mycobacteria and fungi were negative. BAL fluid cytology showed multiple lipid laden macrophages (Figure 3). TBLB showed lung parenchyma infiltrated with foamy macrophages, lymphocytes and occasional neutrophils in the alveolar spaces.

**Figure 4:**
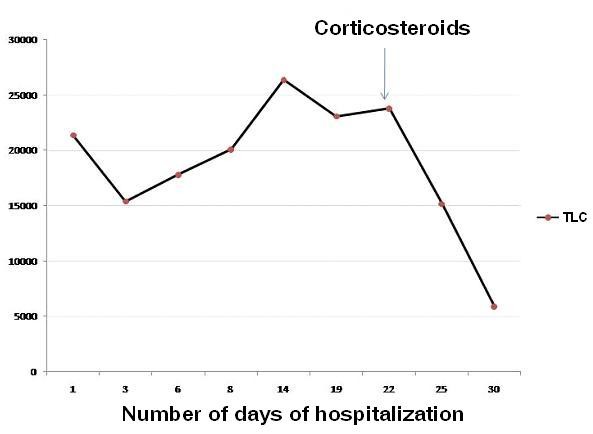
**Line diagram showing TLC response to corticosteroids**.

In view of lipid laden macrophages in BAL and TBLB, we reviewed the history. He recollected that he had aspirated a mineral oil (diesel) during siphoning it from a container, two days prior to onset of illness. So, the diagnosis of exogenous lipoid pneumonia was made. We continued antibiotics and systemic corticosteroids were added in the dose equivalent to 1 mg/kg body weight of prednisolone (initially IV hydrocortisone 300 mg/day and later oral prednisolone 60 mg/day). With this treatment patient's general condition improved. Patient was not requiring oxygen supplementation after a week of therapy. As the TLC started decreasing (Figure 4), IV antibiotics were changed to oral formulations. Patient was discharged on 1 mg/kg body weight of prednisolone for 4 weeks which later was tapered and stopped over next two weeks on out patient basis. After about 2 months of discharge patient was asymptomatic and chest X-ray showed marked resolution (Figure 1b).

## Discussion

Lipoid pneumonia is an unusual cause of respiratory symptoms and has been reported in all age groups. Initial reports of LP predominantly included children - often with local anatomic defects like cleft palate or debilitated adults, but several reports indicate that it can occur in healthy individuals as well [6,7]. There are reports of LP associated with aspirating or inhaling mineral oil, oil based laxatives, lip balm, lip glow etc [7,8]. In our country siphoning of various mineral oils (like diesel) from containers is a common practice and may be an important risk factor for LP. Other risk factors described in infants and small children are use of traditional folk remedies like, use of oily nasal drops, forceful animal fats feeding, such as "ghee", to establish regular bowel habits or transnasal use to treat cough and cold [9,10].

The usual presentation of LP is an insidious onset of dyspnea and/or cough, similar to those of many other chronic lung diseases. Less commonly described clinical features include chest pain, hemoptysis, and intermittent fever [11]. Physical examination findings may be unremarkable or there may be dullness on percussion, crackles, wheezes or rhonchi. Acute presentation simulating infectious pneumonia with fever, with or without cough is unusual [11]. Our patient presented with acute illness with clinical and radiological features consistent with community acquired pneumonia. Blood gas parameters were consistent with acute lung injury (ALI). This is uncommon presentation of LP. However, acute presentation with respiratory failure is reported with accidental/suicidal massive exposure to mineral oil but not with submassive exposure or inhalation [2,3,12].

Laboratory tests are usually normal. However, there may be leukocytosis and an increased erythrocyte sedimentation rate. Radiological findings are diverse and may mimic carcinoma, acute or chronic pneumonia, ARDS, or a localized granuloma [13]. CT scan and magnetic resonance imaging can detect fat within pulmonary tissue. The most commonly described feature is alveolar consolidations of low attenuation values, ground glass opacities with thickening of intralobular septa (crazy paving pattern), or alveolar nodules [13,14]. Pathologically, it is characterized by giant cell granulomatous reaction (hence also called lipid granulomatosis), chronic inflammation, and alveolar and interstitial fibrosis [13]. Diagnosis of LP depends on detecting fat-laden macrophages in sputum, bronchoscopic alveolar lavage (BAL), fine needle aspiration cytology (FNAC) or biopsy from the lung lesion [4,5].

Treatment of LP is not well studied and experiences are only with case reports. Avoiding ongoing exposure and providing supportive care is the main stay of treatment. There are anecdotal reports of systemic corticosteroids use to slow the inflammatory response [15]. However, corticosteroids may not be used routinely and may be used if the lung injury is severe and ongoing. Our case presented with acute lung injury and responded well to corticosteroids. Some authors have described resection of nodules and masses in these cases [9].

Lipoid pneumonia is usually indolent however it may be progressive also. Risk factors for progressive disease are concurrent debilitating illness and continued exposure to mineral oil [7]. Therefore, early diagnosis and avoidance exposure to fatty substances is the most important step to avoid long term sequelae.

## Conclusion

Acute presentation of LP simulating acute CAP with ALI is uncommon. Diagnosis may be delayed like our case where LP was not considered in differential diagnosis until FOB was performed. Therefore, physicians should be aware of this disease which can present acute as well as chronic respiratory illness. Once diagnosed, it may require prolonged corticosteroids for treatment.

## Abbreviations

BAL: bronchoalveolar lavage; FOB: fibreoptic bronchoscopy; IV: intravenous; LP: lipoid pneumonia; TBLB: transbronchial lung biopsy; TLC: total leukocyte count.

## Consent

Written informed consent was obtained from the patient for publication of this case report and accompanying images. A copy of the written consent is available for review by the Editor-in-Chief of this journal.

## Competing interests

The authors declare that they have no competing interests.

## Authors' contributions

VH and GCK were involved in the patient care, acquisition of data, analysis and interpretation of data, review of literature, drafting and revising the manuscript. ASB and SM were involved in reporting of radiological and pathological investigations, respectively. All authors read and approved the final manuscript.
